# Dietary Polyphenols and Obesity

**DOI:** 10.3390/nu2070737

**Published:** 2010-07-08

**Authors:** Mohsen Meydani, Syeda T. Hasan

**Affiliations:** Vascular Biology Laboratory, Jean Mayer USDA- Human Nutrition Research Center on Aging at Tufts University, 711 Washington Street, Boston, MA 02111, USA; Email: sthasan@gmail.com

**Keywords:** polyphenol, green tea, catechins, resveratrol, curcumin, obesity

## Abstract

The prevalence of overweight and obesity and their associated metabolic disorders are considered a major threat to the public’s health. While several diet and exercise programs are available for weight loss and prevention of weight regain, progress is often slow and disappointing. Recently, natural bioactive phytochemicals present in foods have been discovered for their potential health benefit effects on the prevention of chronic disorders such as cancer, cardiovascular disease, inflammatory and metabolic diseases including obesity. Polyphenols are a class of naturally-occurring phytochemicals, of which some such as catechins, anthocynines, resveratrol and curcumin have been shown to modulate physiological and molecular pathways that are involved in energy metabolism, adiposity, and obesity. The potential *in vivo,* beneficial effects of these polyphenols on adiposity and obesity as complementary agents in the up-regulation of energy expenditure have emerged by investigating these compounds in cell cultures, animal models of obesity and in some human clinical and epidemiological studies. In this brief review, the efficacy of the above-named polyphenols and their potential efficacy to modulate obesity and some associated disorders are discussed.

## 1. Introduction

The prevalence of overweight and obesity and associated metabolic complications and related morbidity has increased dramatically in the past two decades [1]. Obesity or body mass index (BMI) > 30 kg/m^2^ is a disease that affects more than 30% of United States adults, with a higher incidence among women. It is expected that one in three children born in the early current century will develop diabetes associated with obesity [1,2]. In addition to diabetes, obesity is a major risk factor for cardiovascular diseases, several forms of cancer (such as breast, colon, and prostate), pulmonary, osteoarticular and metabolic diseases [3,4,5,6,7,8]. It also accounts for 5-7% of the national health expenditure in the United States [9]. Thus, obesity is considered a major threat to the public’s health. Diet and behavioral modification programs for weight loss and the prevention of weight regain aim to reduce energy intake and to increase energy expenditure. However, the ineffectiveness of most approaches is seen in the fact that the prevalence of obesity is at an all-time high and that weight regain is common [10]. Over the past two decades, chemicals derived from plants and known as “phytochemicals” have gained the interest of public and scientific communities for their role in maintaining health and preventing disease. Polyphenols derived from many components of the human diet are among the leading phytochemicals, and some of their potential preventive and therapeutic properties have been studied extensively. In this review, the polyphenols with potential efficacy to modulate obesity and associated disorders are briefly discussed.

## 2. Catechins

Second only to water, tea is one of the most popular beverages and is widely consumed throughout the world. Several forms of tea including green, black, oolong and white teas are prepared from the harvest of leaves from the *Camellia sinensis* plant. The teas differ by their processing and bioactive chemical contents. Green tea has been well-investigated and is recognized by ancient Chinese medicine as having many significant health effects on humans. In recent years, the health benefit effects of green tea are mainly attributed to high concentrations of polyphenols, which are collectively called catechins. Green tea contains five major catechins including: catechin, epicatechin, epicatechin gallate, epigallocatechin, and epigallocatechin gallate (EGCG); the latter comprises more than 40% of the total polyphenolic mixture of green tea catechins. Epidemiological, experimental, and clinical studies have suggested several beneficial effects from consuming green tea: antioxidant and anti-inflammatory activities, cancer and cardiovascular disease prevention properties, and anti-obesity. 

The anti-obesity effect of green tea is mainly attributed to catechins, in particular EGCG, which exhibits its anti-obesity effects through several mechanisms including suppression of adipocyte differentiation and proliferation, inhibition of fat absorption from the gut, and suppression of catechol-o-methyl transferase (COMT), an enzyme that inhibits fatty acid oxidation in brown adipose tissue. The mechanism of action of green tea on fat oxidation is reviewed in [11]. EGCG has been shown to inhibit 3T3-L1 adipocyte proliferation by decreasing levels of phosphorylated ERK1/2, cdk2 and cyclin D1 proteins and cell growth arrest at Go/G1[12] and by inducing apoptosis in mature adipocytes [13]. 

In adipocytes, lipid storage and energy metabolism is tightly controlled. Adenosin monophosphate (AMP) activated protein kinase (AMPK) is the master switch in the regulation of energy metabolism. It is activated in response to an increase in the AMP:ATP (adenosine triphosphate) ratio within the cell, and therefore acts as a sensor for cellular energy regulation. AMPK is a heterodimer protein, which is formed from three subunits:  α,  β and γ [14]. The binding of AMP with AMPK allosterically phosphorylates and activates AMPK [15], which in turn shuts down anabolic pathways and supports catabolic pathways through regulating the activity of several key enzymes of energy metabolism. AMPK inhibits the accumulation of fat by modulating downstream-signaling components. For example, it phosphorylates acetyle CoA carboxylase (ACC) and inhibits activity of this enzyme, down regulates fatty acid synthesis pathways, and prevents fat accumulation. Of particular importance, AMPK, by direct inhibition of HMG-CoA reductase, regulates the cholesterol synthesis pathway in the liver [16]. EGCG has been shown to increase both the expression and phosphorylationof AMPK in 3T3-L1 cells and the phosphorylation of downstream target, ACC, which leads to suppression of esterification of fatty acids to triglyceride and which increases fatty acid oxidation [17].Therefore, through several mechanisms, EGCG and other catechins of tea contribute to the reduction of adipogenesis and prevent the growth and expansion of adipose tissue. 

A lower weight gain and a lower adipogenesis observed in mice fed a high-fat diet supplemented with EGCG was suggested to be due in part to EGCG’s inhibitory effect on pancreatic lipase activity resulting in reduced fat absorption from the gut as indicted by the presence of a high fecal fat content [18,19]. In addition to decreasing fat absorption, several studies have shown that supplementing the diet of mice with EGCG decreases diet-induced adipogenesis and obesity by enhancing fat oxidation [20]. In brown adipose tissue, lipid-metabolizing enzymes are up-regulated by catechins through suppression of COMT, which leads to an increase in norepinephrine with prolonged sympathetic stimulation of thermogenesis along with an increase in adenyl cyclase, lipolysis, and fat oxidation. Therefore, consumption of green tea, which also contains caffeine with its own thermogenesis activity (reviewed in [11]), is regarded to be an effective way to reduce and maintain body weight through increasing fat oxidation and energy expenditure. Administration of green tea extract or catechins not only has been shown to be effective on reduction of weight gain but also has resulted in weight loss as observed in overweight and genetically obese laboratory animals [21]. 

There are several epidemiological and clinical studies showing that consuming tea or green tea extracts reduces body weight and several indices of metabolic syndrome in humans. A cross-sectional human study conducted in Taiwan revealed that habitual drinking of oolong and green tea was associated with low body fat, and a longitudinal study from the Netherlands has shown a lower BMI with the consumption of catechins [22,23]. Although study results on the effect of tea consumption on adiposity and body weight are not consistent, clinical trials have overall shown a beneficial effect from consuming high doses of catechins in tea drinks, which effectively resulted in the reduction of body fat and body weight,  particularly when combined with an exercise regimen [24,25,26,27,28,29]. While the EGCG modulation of fat oxidation is regarded to be an important mechanism by which catechins influence adipogenesis, it is important to note that the high intake of catechins or green tea extract containing very high doses of EGCG causes hepathotoxicity, inflammation of and necrotic damage to the liver [30,31]. The genetic variations between Asian and Caucasian populations might be responsible for differential effects of tea consumption on suppression of COMT activity and lipid metabolism. Tea catechins, in addition to decreasing the risk of Type 2 diabetes by reducing body weight gain, may have a direct beneficial effect on diabetic patients. It has been shown that consumption of green and oolong tea by Type 2 diabetic patients increased glucose uptake by the skeletal muscles through up-regulating glucose transporter 4 (Glut 4) and by decreasing translocation of Glut 4 and insulin levels in adipose tissue [32,33]. 

White tea is another product of the Camellia plant. It is prepared by harvesting buds of tea leaves and by a minimal processing, which preserves high amounts of tea polyphenols such as EGCG and methyl xanthenes (like caffeine). Sohle *et al.* [34] examined the effect of white tea extract on adipogenesis in human subcutaneous pre-adipocytes in culture. They found that incubation of pre-adipocytes with white tea extract containing polyphenols and methylxanthines dose-dependently decreased triglyceride incorporation into adipocytes during adipogenesis. They also reported that white tea extract increased lipolysis-activity in differentiated adipocytes, but the levels of lipolysis was not sufficient enough to explain the decrease in triglyceride incorporation into adipocytes. To elucidate a potential molecular mechanism in this process, Sohle *et al.* examined the effect of white tea extract on a pattern of expression of adipocyte determination and differentiation factor 1(ADD1)/ sterol regulatory element binding proteins (SREBP) -1c, a transcription factor for adipocyte differentiation, which also promotes proxisome proliferator-activated receptor (PPAR)γ expression [35]. PPARγ is the master regulator of adipogenesis [36,37]. Using immunofluorescence microscopy, they observed that extract of white tea suppressed ADD1/SREBP-1c signals, which was associated with decreased levels of PPARγ as well as CCAAT/enhancer binding protein (C/EBP)α and C/EBPδ mRNA levels during adipogenesis, suggesting that it may exert its effect through increasing lipolysis and by inhibiting adipogenesis. They noted that when they used human visceral pre-adipocytes, neither white tea extract nor EGCG could reduce triglyceride accumulation. They also reported that the effect of white tea extract in the reduction of adipogenesis is not through modulation of SIRT 1 (silent information regulation 2 homolog 1) levels which is known to modulate cellular metabolism. SIRT 1 is one of the 7 enzymes in the sirtuins family of enzymes [38], and it is known to regulate adipogenesis through inhibiting genes that are involved in adipocyte differentiation and triglyceride accumulation [39]. 

## 3. Anthocyanins and Blueberries

Blueberries are fruits from the *Vaccinium* plant. The fruits are rich in phenolic compounds such as hydroxycinnamic acids, flavonoids, and proanthocyanidines [40,41] and more than 20 anthocyanins [42]. Several experimental animal studies have suggested that the consumption of blueberries, or its bioactive polyphenolic contents with potent antioxidant activities, may provide several health benefits including improvement in cognitive function [43] , antioxidant effects [44], protection against inflammation [45], and modulation of obesity and adiposity. Interestingly, supplementing a high fat diet of C57BL/6J mice (60% calories from fat) with 2.9 mg/g purified anthocyanins extracted from blueberries decreased their body and adipose tissue weight compared to high fat-fed controls, whereas supplementing the high fat diet (45% calories from fat) of mice with 10% freeze-dried extracts of whole blueberries resulted in the increase of body weight gain rather than decreasing it [46]. Moreover, the administration of purified anthocyanins from blueberries also lowered serum triglycerides, cholesterol and leptin levels. Liver lipids and triglycerides levels, however, were not altered [47]. Food intake in these studies was monitored, and the authors reported no significant effect of either blueberry extract or anthocyanins from blueberry on food intake. In a most recent study [48], 10% freeze-dried blueberry powder or 1.16 mg/mL purified anthocyanins from blueberry did not significantly reduce body weight gain compared to high fat-fed control mice, however mice fed purified anthocyanins from blueberries had a lower percent of body fat. Notably, administration of anthocyanine to the high fat-fed mice also altered several obesity-associated parameters including fasting blood glucose and leptin levels and enhanced β-cell function. Blueberry juice supplementation, however, did not change these parameters except for lowering leptin levels. Leptin is a hormone produced by adipocytes [49,50], which reduce triglycerides formation in various organs by increasing free fatty acid (FFA) oxidation and decreasing its esterification to triglyceride; thus, it reduces insulin resistance and β-cell dysfunction, which is known to lead to obesity and associated diabetes [51]. At present, it is not known why and how anthocyanins from blueberries, but not blueberry extract or juice, reduce adiposity and associated parameters. It was suggested that the absorption and bioavailability of anthocyanins from the gut might be hindered by the presence of sugars, carbohydrates, and lipids in the whole blueberry extract [48].

Biotransformation of blueberry juice by bacteria (*Serratia vacinii)* has been shown to increase the phenolic content of blueberry juice and to quadruple its antioxidant activity. When administered to KKA^y^ mice (40 mL·kg^-1^/day, for three weeks), a model of obesity and diabetes, the biotransformed juice reduced body weight gain,  abdominal fat pads, and liver weights possibly through its anorexic effect. However, when biotransformed juice was chronically administered (80 mL·kg^-1^/day), no reduction in body weight gain was observed, but it reduced blood glucose levels, tended to reduce blood insulin levels, and increased adiponectin levels in diabetic mice [41]. Adiponectin is an adipocytokine and has been shown to reverse insulin resistance in obese mice. Adiponectin also lowers muscle triglyceride levels by increasing influx and combustion of free fatty acids resulting in a decreased hepatic level of triglycerides [52].

In another study, supplementing a high fat diet (60% calories from fat) of C57BL/6J mice with a 4% freeze-dried, whole blueberry powder showed no attenuation of weight gain or adipose tissue weight. However, freeze-dried whole blueberry increased insulin sensitivity and improved glucose homeostasis in mice [53]. Adipocyte death in adipose tissues is one of the phenomena that occur with diet induced obesity. The dead adipocytes attract macrophages to the adipose tissue resulting in release of proinflammatory cytokines like TNF-α, IL-6 and MCP-1. The chronic production of these inflammatory cytokines can lead to the development of insulin resistance [31,42,46,54]. Mice supplemented with 4% blueberry extract showed a protection against adipocyte death along with a down-regulation in macrophage gene expression of TNF-α and IL-10. The gene expression of CD11c as a surface marker for macrophages was also down regulated indicating an inhibition of macrophage infiltration [53]. Although the effects of blueberry extracts in different studies do not indicate a beneficial effect on obesity, it was found that the parameters affected by obesity such as insulin sensitivity and inflammation are attenuated by blueberry supplementation.

## 4. Resveratrol

Resveratrol is a well-studied polyphenol, present in red grapes, red wine, peanuts, and ground nuts. It has antioxidant and anti-inflammatory properties [55] and has been suggested to be effective in preventing the development of several diseases including cardiovascular disease, diabetes, cancer, and obesity [56,57,58]. Resveratrol’s beneficial effects on obesity are reported to be due in part to its ability to increase the phosphorylation and activation of AMPK, the master regulator of energy metabolism, which up-regulates fatty acid oxidation and increases uptake of glucose through Glut 4 translocation [59]. Resveratrol has also been suggested to increase glucose uptake through up-regulation of estrogen receptor-α, which in turn increases Glut 4 expression through phosphatidyle inositol-3 kinase (PI3K) and AKT pathway. Furthermore, through up-regulation of SIRT 1, resveratrol increases PPARγ coactivator (PGC)-1α leading to mitochondrial biogenesis, oxidative phosphorylation, and thus contributes to the suppression of lipid accumulation [60,61]. Ahn *et al.* [60] found that resveratrol supplementation also increased the liver’s expression of SIRT 1 and suppressed expression of PPARγ, accumulation of fat in liver of mice fed high fat atherogenic diet. Floyd *et al.* [61] have shown that treatment of 3T3-L1 cells with resveratrol reduced PPARγ expression in part through ubiquitin-dependent proteasome degradation. The addition of resveratrol to rat isolated hepathocytes has also been shown to inhibit fatty acid and triglyceride synthesis; thus, it contributes to resveratrol’s lipid lowering effect [62]. Cell culture studies also revealed that resveratrol enhances lypolitic activity in adipocytes through induction of cAMP [63] and inhibits adipogenesis in isolated human adipocytes. These effects of resveratrol have been shown to be potentiated when combined with genistein [64,65]. Thus, resveratrol, through these metabolic effects, may exert several beneficial effects in the prevention of obesity and diabetes. Indeed, several laboratory animal studies have shown that resveratrol treatments significantly reduced fat depots size and total body fat in high fat fed and genetically obese rodents. The treatment of rats consuming a hypercaloric diet with 30 mg resveratrol per kg b.w. for 6 weeks reduced total adipose tissue [66] and reduced visceral fat and liver mass indices in rates fed a high fat diet [67]. In obese Zucker rats, the administration of resveratrol was not as effective as with regular rats fed a high fat diet, but it reduced plasma triglycerides, free fatty acids, cholesterol, and liver triglycerides [68]. Giving resveratrol to mice fed a high fat diet diminished total body fat content and decreased epididymal, inguinal, and peritoneal adipose tissues [60,69]. Mice fed a high fat-atherogenic diet supplemented with 125 mg resveratrol/kg diet gained less body weight and accumulated less total fat and triglyceride; further, they had a lower liver weight compared to non-supplemented mice [60]. The microarray analysis of genes in the liver revealed that resveratrol supplementation down-regulates the expression of genes involved in lipogenesis. In animal models of diabetes, resveratrol reduced blood insulin levels and hyperglycemia (reviewed in [70]). Through its antioxidant activity, resveratrol may also prevent oxidative damages resulting from impaired glucose metabolism, and thus may prevent the pathogenesis of diabetic complications. Several lines of evidence also indicate that administering resveratrol to rodent models induces the kind of biological changes comparable to those observed in caloric restriction, such as an increase in longevity and motor functions, as well as prevention of cardiac and skeletal muscle dysfunction associated with aging [71]. Resveratrol, through its antioxidant and anti-inflammatory properties, has been shown to protect against liver damage induced by hepathotoxins (reviewed in [72]). No adverse effects of resveratrol have been reported in rabbits and rats, and only mild effects in a few cases were reported in human studies [54]. However, very high doses of resveratrol (3,000 mg/kg b.w./day) (<<<repeats later in sentence) in one study resulted in anemia and nephrotoxicity within 4 weeks in female rats showing a high sensitivity to resveratrol toxicity[73]. This level of high dose resveratrol also showed toxicity to the liver as determined by high levels of alanine transaminase, alkaline phosphatase, and total bilirubin [73,74]. In another study, old mice treated with 14.09 mg resveratrol/L in drinking water had increased oxidative damage in the kidneys as measured by 8-hydroxy-2’-deoxyguanosine [75]. Several human clinical studies have examined the bioavailability of resveratrol using doses between 25 mg per person to 5 g per person. A Phase I, dose-escalation pharmacokinetic study using 0.5-5 g per healthy volunteer did not exhibit adverse effects in humans. 

While compelling evidence from molecular, cell culture, and animal studies suggest that resveratrol potentially contributes to the prevention of obesity through multiple mechanisms, epidemiological or clinical studies are needed to support whether the consumption of resveratrol is also effective in preventing obesity in humans. 

## 5. Curcumin

Curcumin is the major, bioactive polyphenol present in the spice turmeric, which is the ground rhizome of the perennial herb *Curcuma longa*. In addition to being used as a spice and colorant, turmeric has been used in Asian medicine since the second millennium BC [76]. Curcumin is a low molecular polyphenol with several biological properties. It has been shown to possess antioxidant, anti-inflammatory, anticancer, anti-angiogenesis, chemopreventive and chemotherapeutic properties [77]. The first report referring to curcumin’s effect on disease in humans was published in *The Lancet* about 80 years ago [78]. Rao *et al.* reported that curcumin supplementation at the dose of 500-1,000 mg/kg in rat diet reduced liver cholesterol and increased bile acid excretion [79]. At a dose of 250 mg/kg, curcumin was also reported to reduce weight gain in rats after 4 weeks and tended to reduce liver weight as well as blood triglyceride and free fatty acids levels [80].

In addition to the above-mentioned earlier studies, recent cell culture and animal studies have explored the impact of curcumin on lipid metabolism, adiposity, and inflammation in more detail. Curcumin may have a significant effect on adiposity and lipid metabolism through several mechanisms including modulation of energy metabolism, inflammation, and suppression of angiogenesis. It has been well established that angiogenesis plays pivotal roles in the growth and expansion of adipose tissue (reviewed in [81,82,83,84]) was also reported to reduce weight to suppress angiogenesis, it has mainly been investigated for its effect on cancerous tumor growth. However, it may play an important role in the growth and expansion of adipose tissue as well. It is known that through down-regulation of several factors including vascular endothelial growth factor (VEGF), basic fibroblast growth factor (bFGF) and epidermal growth factor (EGF), as well as angiopoietin and hypoxia-inducible factors (HIF)-1α, curcumin suppresses angiogenesis and restricts the growth of tumors [85,86]. Therefore, curcumin may contribute to the prevention of adipogenesis through suppression of angiogenesis into the adipose tissue [87]. In adipose tissue, angiogenesis is mediated by adipose tissue secretion of adipokines including leptin, adiponectin, resistin, visfatin, tumor necrosis factor (TNF)-α, interleukin(IL)-6, IL-1, and VEGF [88]. Therefore, the inhibition of angiogenesis in adipose tissue can be used as a strategy to prevent the growth of adipose tissue and thus, obesity. We have demonstrated this effect of curcumin in our recent study [89] where supplementing a high fat diet of C57BL/6J mice with curcumin reduced microvessel density as an indication of suppression of angiogenesis in adipose tissue.

Like other polyphenols reviewed above, we in our recent study found that curcumin activated AMPK and down-regulated ACC activity through phosphorylation of this enzyme, which in turn down-regulated the flow of acetyl CoA to malonyl CoA leading to up-regulation of carnitine palmitoyltransferase-1 (CPT-1), which transfers cytosolic long-chain fatty acyl CoA into the mitochondria for oxidation [90]. In addition, through activation of AMPK, curcumin down-regulated synthesis of glycerol lipids by inhibiting glycerol-3-phosphate acyl transferase-1 (GPAT-1) activity, which esterifies fatty acids to glycerol to form triglycerides for storage [89]. 

These effects of curcumin on energy metabolism were observed both in adipocyte cultures and in adipose tissue of mice fed a high fat diet. Several other studies in animal models of obesity have reported the beneficial effects of curcumin on body weight and fat, adiposity, and energy metabolism. Asai and Miyazawa [91] reported that relatively high dietary curcumin supplementation (2 and 10 g/kg diet) for two weeks in rats reduced epididymal adipose tissue, attenuated liver fatty acid synthesis, and increased rat liver acetyl CoA oxidase activity, which is the first catalytic enzyme in fatty acid β-oxidation. Recently, it was reported that supplementing the high fat diet of hamsters with 500 mg/kg curcumin reduced the levels of free fatty acid, total cholesterol, triglycerides, and leptin as well as the insulin resistance index [92]. The hypoglycemic effect of ethanolic extract of turmeric has also been reported in genetically diabetic KK-Ay/Ta mice [93]. Jang *et al.* also reported that in hamsters fed a high fat diet, curcumin supplementation increased the hepatic β-oxidation and decreased fatty acid and cholesterol synthesis [92]. These observations are in support of our findings that curcumin supplementation suppressed a high fat diet-induced fatty liver in mice and reduced plasma levels of cholesterol, triglycerides, glucose, and free fatty acids. In a mouse model of insulin resistant obesity, Weisberg *et al.* [94] recently reported that inclusion of a generous amount of curcumin in the diet significantly ameliorated type 2 diabetes and inflammation in the liver as detected by a lower expression of nuclear factor (NF)-κB and reduced the infiltration of macrophages in adipose tissues. They also reported that although mice consumed more curcumin-supplemented food (*i.e.,* more calories), they nevertheless displayed a lower body weight and a lower body fat as measured by nuclear magnetic resonance (NMR). 

In our study, [89] we also found that curcumin supplementation suppressed expression of PPARγ and C/EBPα, transcription factors that are mainly found in adipose tissue and are the key transcription factors in adipogenesis and lipogenesis [95]. Curcumin also suppressed differentiation of pre-adipocytes to adipocytes, which in turn attenuated adipose tissue growth and expansion. This effect of curcumin might have been mediated through suppressing the expression of PPARγ transcription factor because a PPARγ agonist, such as thiazolidinedione, induces differentiation of human pre-adipocytes and increases subcutaneous adiposity [96]. Therefore, suppression of these transcription factors by curcumin is another potential mechanism by which curcumin contributes to the suppression of adipogenesis. 

It is worth mentioning that studies on the effect of curcumin on metabolic syndrome and obesity have been conducted in the experimental animals, whereas supplementation studies on humans are mainly limited to investigations related to curcumin’s anti-inflammatory and anti-cancer properties. Therefore, curcumin’s anti-obesity effect in humans remains to be demonstrated after establishing its safety after long-term use.

## 6. Concluding Remarks

Cell culture, animal, and limited human studies suggest that consumption of foods containing certain polyphenols or their corresponding supplements changes lipid and energy metabolism and may facilitate weight loss and prevent weight gain. Evidence from pre-clinical and some clinical studies indicates that consumption of green and white teas containing catechins, fruits such as blueberries with anthocyanins, foods such as red grapes and wine with resveratrol, and spice like turmeric containing curcumin may provide several health benefits including improving blood glucose and lipid profiles, ameliorating insulin resistance, adiposity and obesity. Current knowledge suggests that the potential complementary effect of these polyphenols may occur through several mechanisms: suppression of fat absorption from the gut, uptake of glucose by skeletal muscles, suppression of anabolic pathways, stimulation of catabolic pathways in adipose tissues, liver and other tissues, inhibition of angiogenesis in adipose tissues, inhibition of differentiation of pre-adipocytes to adipocytes, stimulation of apoptosis of mature adipocytes, and reduction of chronic inflammation associated with adiposity. It is important to note that high doses of these polyphenols in supplement form may have adverse effects. At present, there is not sufficient data to support recommending long-term, safe usage of these polyphenols for prevention and treatment of obesity. Nevertheless, including foods containing these polyphenols in the diet following the US dietary guideline for healthy eating and exercise may help to prevent obesity and to maintain an ideal body weight.
